# Mortality Characteristics of Two Populations in the Northern Mediterranean (Croatia) in the Period 1960–2012: An Ecological Study

**DOI:** 10.3390/ijerph15112591

**Published:** 2018-11-20

**Authors:** Robert Doričić, Tanja Ćorić, Morana Tomljenović, Danijela Lakošeljac, Amir Muzur, Branko Kolarić

**Affiliations:** 1 Faculty of Medicine, University of Rijeka, Rijeka 51000, Croatia; Tanja.Coric@stampar.hr (T.Ć.); morana.tomljenovic@medri.uniri.hr (M.T.); amir.muzur@medri.uniri.hr (A.M.); Branko.Kolaric@stampar.hr (B.K.); 2 Andrija Štampar Teaching Institute of Public Health, Zagreb 10000, Croatia; 3 Teaching Institute of Public Health of Primorje-Gorski Kotar County, Rijeka 51000, Croatia; danijela.lakoseljac@zzjzpgz.hr; 4 Faculty of Health Studies, University of Rijeka, Rijeka 51000, Croatia

**Keywords:** Mediterranean, Croatia, Bakar, Mali Lošinj, air pollution, mortality, diseases of the respiratory system, COPD, neoplasms, stomach carcinoma

## Abstract

In the second half of the 20th century, the town of Bakar (Primorje-Gorski Kotar County, Croatia), where a coking plant was operational 1978–1994, experienced intensive industrialisation. The town of Mali Lošinj (Primorje-Gorski Kotar County, Croatia) in this period based its economy on non-industrial sectors. The study goal was comparing mortality characteristics of these populations in the northern Mediterranean for 1960–2012. An ecological study design was used. Data were analysed for 1960–2012 for the deceased with recorded place of residence in the study area. Data on the deceased for 1960–1993 were taken from death reports, for 1994–2012 from digital archives of the Teaching Institute of Public Health, Primorje-Gorski Kotar County. Data on causes of death for 1960–1994 were recoded to the three-digit code of underlying cause of death according to the International Classification of Diseases (ICD–10). Among studied populations significant difference was found among the causes of deaths coded within ICD–10 chapters: neoplasms (particularly stomach carcinoma), mental and behavioural disorders and diseases of the respiratory system (particularly chronic obstructive pulmonary disease, (COPD)). Increase in mortality from neoplasms, increase in respiratory diseases for the area exposed to industrial pollution, also stomach carcinoma and COPD particularly in the town Bakar require further research.

## 1. Introduction

**Figure 1:**
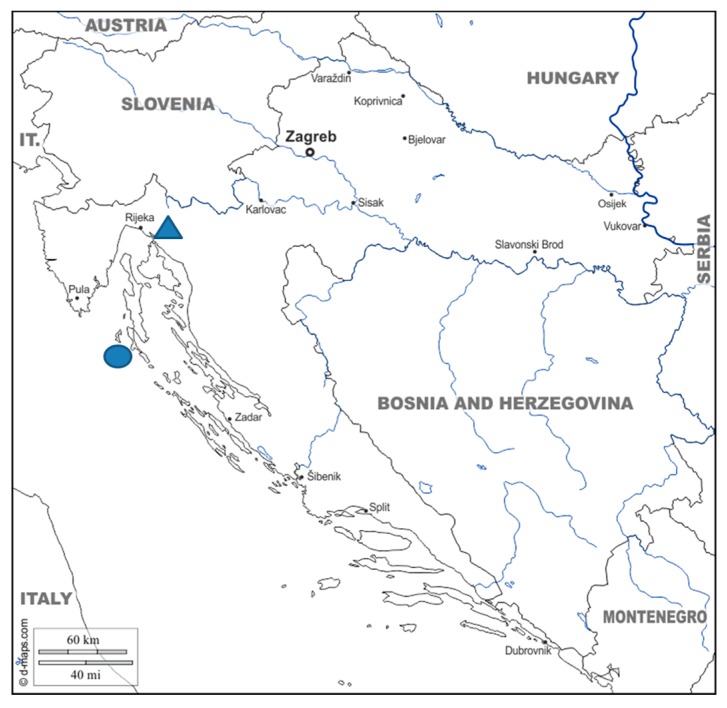
Location of the two studied populations (▲ = town of Bakar; ● = town of Mali Lošinj). Background map source: https://d-maps.com/carte.php?num_car=14898&lang=en.

The towns of Bakar and Mali Lošinj in the Primorje-Gorski Kotar County of the Republic of Croatia are two administrative units in the Northern Adriatic area and are part of the Littoral Croatia, one of the three natural-geographical parts of Croatia (Figure 1) According to geomorphological regionalisation they are a part of the macro-geomorphological region of the Istrian Peninsula with the Kvarner Littoral and Archipelago [1]. Climatologically, this area, aside from the most southern part of the Lošinj region, falls into the C category of moderately warm rainy climate [2].

The centre of the administrative unit of the town of Bakar is the settlement of the same name—Bakar, located on the north-eastern edge of the Bay of Bakar, 7.6 km airway distance to the south-east of the city of Rijeka—the macro-regional centre and the centre of the Primorje-Gorski Kotar County. In the first decades following the Second World War, in the wider area of the Rijeka Ring—the area surrounding the city of Rijeka, a process of intensive industrialisation took place. In the Bay of Bakar several power and industrial plants were built. Thus in 1965 the Urinj oil refinery started operating followed by the thermal power plant Urinj and the coking plant Bakar in 1978 [3]. Depending on the leading economic sector, the structure of the employed population in the area of the town of Bakar lived to see several changes in the period 1960–2012. According to the 1961 census, the majority of the employed persons with a residence in the town of Bakar were employed in the tertiary sector notably in the traffic and trade industries while according to the 1971 and 1991 census the primacy is taken over by the secondary sector (industry) following which the tertiary sector again takes the first place [4,5,6,7,8].

The coking plant, which operated in the period from 1978 to 1994 in the immediate vicinity of the settlement of Bakar, was one of the most significant polluters of this area [9,10,11]. At the monitoring station in the Bakar town area, the average annual concentrations of sulphur dioxide (SO_2_) (50 μg/m^3^) were found to be above the recommended levels [12]: at the monitoring station Bakar (0.6 airway distance from the coking plant) in the period 1980–1994, at the monitoring station Krasica (1.5 km airway distance from the coking plant) in the period 1983–1993, and at the monitoring station Kukuljanovo (3.5 km airway distance from the coking plant) in 1979, followed by the periods 1985–1990 and 1992–1993. At all three monitoring stations, only in 1984 no elevated annual levels of SO_2_ were found (Figure S1).

In this period, studies were conducted with the goal to investigate the relation of environmental pollution in the wider Bakar Bay area and its impact on the health status of the population [9,13]. A study conducted in 1981–1982 among pre-school children in the wider area of the town of Rijeka included the settlements of Bakar and Krasica. A positive correlation was determined between air pollution and acute respiratory illness in pre-school children [11]. Cross-sectional epidemiological studies of chronic respiratory disease prevalence among the domicile population were conducted in 1986 and 1990. In this period, mean annual SO_2_ concentrations at the measuring station Bakar were highest in the first (1986) and the last (1990) year of study (74 μg/m^3^) and the lowest in 1988 (67 μg/m^3^). Heightened mean annual SO_2_ concentrations were measured in 1987 and 1989 (72 μg/m^3^). The relation of SO_2_ concentration in the air and the prevalence of chronic bronchitis among groups of women in the settlements of Bakar and Krasica was investigated in comparison to the non-exposed group of women from the municipality of Viškovo, a settlement situated to the north-west to the city of Rijeka in which there were no industrial plants. Research results among the population of women in Bakar and Krasica conducted in 1986 showed that a higher presence of obstructive changes in the respiratory system among women from Bakar and Krasica was not statistically significant in relation to their presence among the non-exposed group from Viškovo. In this study, the first etiological factor for the development of chronic bronchitis smoking was noted [13]. A study conducted in 1990 among groups of study participants from Bakar, Krasica and Viškovo noted the assumption on the relation of long-term exposure to air pollution and lung function disorders among study participants from Bakar and Krasica. The measured forced vital capacity (FVC), forced expiratory volume in one second (FEV1), values of FEV1/FEV ratio, i.e., Tiffeneau index (TT) and values of maximal expiratory flow between 25% and 75% of expired vital capacity (FMF 25–75%) at both measurements was lower in relation to the control group from Viškovo [9]. Towards the end of the 1980s, studies were conducted among the population of employees in the coking plant in Bakar. Subjective visual disturbances among employees were confirmed by a result of the changes to the anterior segment of the eye with a high percentage. Among employees working at the coking plant for less than two years, there were no changes in tear secretion, while among those working at the coking plant between 2 and 7 years, hypersecretion has been determined, and among those employed for more than 7 years hyposecretion has been determined. This study noted the probability of the relation of ophthalmic disturbances to the working conditions [14]. Despite their limitations, these studies represent the first investigations into the influence of environment polluters on the health of the population in the Bakar Bay area in the second half of the 20th century.

The area of the town of Mali Lošinj covers the southern part of the Island of Cres and the islands of the Lošinj archipelago. This is also the most southern part of the Primorje-Gorski Kotar County, Croatia. The centre of the administrative unit is the town of Mali Lošinj, situated 88.6 km of airway distance to the southwest from the city of Rijeka. Already towards the end of the 19th century, the island of Lošinj has, due to its microclimatic characteristics, been proclaimed a climate resort and rehabilitation center [15], from the 1960-ies, alongside the health tourism, mass tourism also started developing [16,17]. According to the 1961 census data, the largest proportion of the active population made its income in agriculture; in the following decades the structure of employment changed, and primacy has to this day stayed with tertiary activity [4,5,6,7,8,18]. The area of the town of Mali Lošinj has in the entire researched period not been subject to significant pollution caused by the steel and metal industry or other larger sources of industrial pollution. Even though there is no systematic air-quality monitoring in the entire area of the town of Mali Lošinj, periodic measurements conducted in the town of Veli Lošinj in the periods 1986–1992, and 2007–2008 have shown that the air quality in this area was of I. category [19,20].

The overview of changes to the size of the population in the researched area (Table 1) points to a significantly negative bearing of changes in the period between censuses 1961–1971 for the area of the town of Mali Lošinj. Such a change was a result of depopulation in the settlements of the Lošinj islands [16,21]. The town of Bakar area has shown a positive demographic movement in spite of depopulation recorded in the two periods between censuses (1971–1981; 1991–2001) [22,23].

In the period of significant dynamics in economical trends, interesting particularly from the comparative perspective, there is an evident shortage of scientific and expert articles on mortality structure for the area that today falls under the administrative units of the towns of Bakar and Mali Lošinj. An exception to this is a systematic review of mortality structure for the parish of Bakar in the 19th century [30,31].

The goal of this study is to describe and compare mortality characteristics in the period 1960–2012 for the populations from the areas of the towns of Bakar and Mali Lošinj, Primorje-Gorski Kotar County, Croatia.

The only relevant dataset covering the entire study period (1960–2012) and based on which an analysis of features of deaths is possible, are data on causes of deaths. Namely, the existing hospital data system at the country level has not been digitalised and does not enable the use of any prevalence indicators.

## 2. Materials and Methods

This research has the characteristics of an ecological study. Data on deaths in the period from 1 January 1960 to 31 December 2012 were analysed for persons whose last place of residence was registered in the administrative units of the towns of Bakar and Mali Lošinj. Comparing the number of residents in the two administrative units according to census years (Table 1), we conclude that, according to the number of residents, these are two similar populations. A significant difference in the numbers of residents was determined only in data for census years 1971 and 1991. Data on deaths for the period from 1 January 1960 to 31 December 1993 were extracted from the reports on the deaths kept in the archives and outposts of the State Administration Office in Primorje-Gorski Kotar County in Bakar and Mali Lošinj. Archive records kept at the Teaching Institute of Public Health of Primorje-Gorski Kotar County were also used. There is an artefact deviation for the town of Mali Lošinj for the years 1972, 1975 and 1976 due to missing parts of the archive records. Even though the number of deaths included in the sample for the area of Mali Lošinj is smaller for the mentioned years than the average annual number of deaths, these data were included in the total study sample.

Analysed data included the following demographic variables of the deceased: gender, age and last recorded place of residence. In the area of the town of Mali Lošinj until the end of the 19th century there was a home for the aged and infirm. In order for those who died at that home to be included in the study sample, at least one of the two criteria had to be met:

1. Place of residence before institutionalisation had to be the town of Mali Lošinj;
2. Place of birth was the area of the town of Mali Lošinj.

Causes of deaths were extracted from the entire documentation. Data on deaths for both studied populations for the period 1 January 1994 to 31 December 2012 were taken from digital archives of the Teaching Institute of Public Health of Primorje-Gorski Kotar County. These data include demographic variables (gender, age and last recorded place of residence) and the coded cause of death. Since data were collected in the time period in which several systems of determining and coding the underlying cause of death were used [32,33], data on the cause of death for the period 1 January 1960 to 31 December 1994 were recoded to the three-digit code of the underlying cause of death in line with the current regulations and recommendations of the Department for Mortality Statistics of the Croatian Institute of Public Health for coding the underlying cause of death [34].

The total studied period was divided into three sub-periods: 1960–1977, the period before the coking plant in Bakar was opened; 1978–1994, the period in which the coking plant was operational; and 1995–2012, the period after the coking plant was closed.

General mortality rate for the period 1960–2012 was analysed in both studied populations (Tables S1 and S2).

Using the direct standardisation method, a standardised mortality rate according to age was determined (Tables S3 and S4) [35]. Age standardisation was done according to the world standard population [36,37]. For the age structure of the studied populations, census data from 1961–2011 were used. Standardisation according to gender was not possible due to the fact that for the period 1961–1971 gender distribution in the mentioned populations was not known, while for the period 1972–1981 data were incomplete. Standardised mortality rates of studied populations for the period 1985–2012 were compared to the age-standardised rates at the Croatian level. Namely, in the digital World Health Organization (WHO) database [36] mortality data are available from 1985 for federative republics of the former Socialist Federative Republic of Yugoslavia an integrative part of which, until 1991, was also present-day Republic of Croatia. Due to a small number of the deceased by causes from a single group of diseases, causes of deaths were aggregated into five-year age groups in both studied populations (0–4, 5–9, 10–14 all up to 65+) [24,25,26,27,28,29].

For the leading causes of death classified according to the chapters of the International Classification of Diseases (ICD–10), with the exception of the chapter *Symptoms, signs and abnormal clinical and laboratory findings, not elsewhere classified* (chapter XVIII, ICD–10) average age-standardised mortality rates for the three studied time periods were determined. For both studied populations ten leading causes of deaths were found.

Average age-standardised rates were also determined for individual leading causes of deaths. Individual leading causes of deaths were chosen according to two criteria:

1. Belonging to leading causes of deaths classified according to ICD–10;
2. Inclusion among 10 leading causes of deaths of individual studied populations.

Presentation of annual mortality data for the Republic of Croatia includes a presentation of proportionate mortality; this was the reason for its inclusion in this study, which will enable a comparative analysis.

### 2.1. Statistics

Distribution of numerical data variables was tested using the Kolmogorov–Smirnov test. Comparison of categorical variables was assessed using the χ2 or Fischer’s exact test. Ordinal variables were compared using the Mann–Whitney U test. The level of statistical significance was set as α = 0.05.

Statistical analysis was performed using STATA/IC ver 14.1 (StataCorp LP, College Station, TX, USA).

### 2.2. Research Ethics

The study was conducted in accordance with the Declaration of Helsinki, and the protocol was approved by the Ethics Committee of the University of Rijeka, Faculty of Medicine (003–08/17–01/19).

## 3. Results

The total sample size was 8565 death records (Bakar: 4472; Mali Lošinj: 4093) (Table S1) of persons who have died in the period 1960–2012. According to gender distribution, in the town of Bakar a total of 2134 (47.72%) men and 2338 (52.28%) women have died, while in the town of Mali Lošinj 1879 men (45.93%) and 2212 (54.07%) women have died. In both populations more women have died. Based on available death records data, gender could not be determined for two death records in the age group 0–4, included in the sample in the population from the town of Mali Lošinj.

A statistically significant difference was found in the age of the deceased from the town of Bakar compared to the area of the town of Mali Lošinj, the median age of the deceased from the town of Bakar was 76 years (66–83), which is lower compared to the median age of the deceased from the town of Mali Lošinj 78 (68–85) (*p* < 0.001).

A statistically significant difference was also found when comparing the deceased according to gender in each studied population. In the Bakar town area, the median age of deceased women was 80 years (71–86), while the age of the deceased men was 71 years (61–80) (*p* < 0.001). The median age of deceased women in the area of the town of Mali Lošinj was 81 years (72–86), while the median age of the deceased men was 74 years (62–82) (*p* < 0.001). In both populations women have died at an older age compared to men.

### 3.1. General Mortality

In both populations studied an average mortality rate was analysed in three studied sub-periods. In the first studied sub-period 1960–1977, mortality in the area of the towns of Bakar and Mali Lošinj was approximately similar (town of Bakar 1060.92/100,000, town of Mali Lošinj: 1056.63/100,000). Larger deviations were recorded in the second and third studied sub-period in comparison to the first studied sub-period. In the town of Bakar, the average mortality rate increased compared to the first studied sub-period. In the second studied sub-period, it reaches the value of 1092.11/100,000 and in the third 1083.54/100,000. In the town of Mali Lošinj, the average mortality rate in the second and third studied sub-period has decreased, in comparison to the first studied sub-period and is almost the same in the second and third studied periods (in the second 944.83/100,000, and in the third 947.78/100,000).

### 3.2. Age-Standardised Mortality Rate

**Figure 2:**
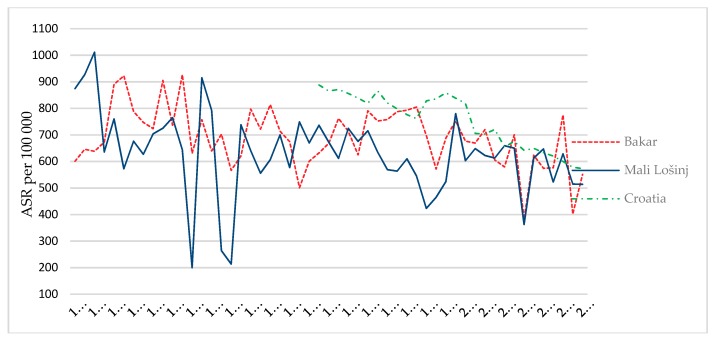
Age-standardised mortality rate in the towns of Bakar and Mali Lošinj in the period 1960–2012/100,000 population.

Age-standardised mortality rate (Figure 2) for the area of the town of Bakar shows a growing trend from the first half of the 1960s (Table S1). In the mid 1980s, in the period 1983–1986, age-standardised mortality rates for the area of the town of Mali Lošinj were higher than those for the town of Bakar. In the last studied decade in both studied populations the age-standardised rates found were higher compared to the age-standardised mortality rate for the level of the Republic of Croatia. Age-standardised mortality rates in the areas of both towns in 2010 are higher than age-standardised mortality rates at the level of the Republic of Croatia. Age-standardised death rate for the area of the town of Bakar is higher than the age-standardised rate for Croatia in 1995, 2002, and 2005. Age-standardised rate for the area of the town of Mali Lošinj is higher than that for the Republic of Croatia for 2004 and 2008.

### 3.3. Causes of Death According to the International Classification of Diseases (ICD–10) Groups

In the towns of Bakar and Mali Lošinj in the total studied period, the leading causes of death (Table 2) are *Diseases of the circulatory system* (chapter IX, ICD–10). The share of the causes of deaths pertaining to this chapter were at 48.19% and 46.57% for the towns of Bakar and Mali Lošinj, respectively. *Neoplasms* (chapter II, ICD–10) (Table S5) take the second place. Their share in the total sample amounted to 22.43% and 18.59% for the towns of Bakar and Mali Lošinj respectively.

For the town of Bakar, the causes of death from chapter *External causes of morbidity and mortality* (chapter XX, ICD–10) are in the third place with a share of 6.04%, while diseases from chapter *Mental and behavioural disorders* (5.14%) (chapter V, ICD–10) (Table S6) take the fourth place. In the town of Bakar area, diseases from chapter *Diseases of the respiratory system* (chapter X, ICD–10) (Table S7) take the fifth place with a share of 4.03%.

In the town of Mali Lošinj, causes from the group *Symptoms, signs and abnormal clinical and laboratory findings, not elsewhere classified* (chapter XVIII, ICD–10) are ranked in the third place with a share of 8.48%. Causes of death from chapter *Diseases of the respiratory system* (chapter X, ICD–10) take the fourth place (7.48%), while the fifth place is occupied by causes of death from chapter *External causes of morbidity and mortality* (chapter XX, ICD–10) with a share of 5.18%.

In the total sample including both genders, causes of death from chapter *Symptoms, signs and abnormal clinical and laboratory findings, not elsewhere classified* (chapter XVIII, ICD–10) are present with a share of 5.88%.

Comparison of deaths caused by neoplasms (chapter II, ICD–10) (Table 2 and Table S5) in the population of the town of Mali Lošinj shows an increase in the studied sub-periods while in the town of Bakar, the number of deaths in the second studied sub-period has decreased compared to the first and third sub-period. The number of causes of deaths from chapter *Mental and behavioural disorders* (chapter V, ICD–10) in the first two studied sub-periods is significantly higher in the town of Bakar, compared to the number of deaths in the town of Mali Lošinj. Causes of deaths from the chapter *Symptoms, signs and abnormal clinical and laboratory findings, not elsewhere classified* (chapter XVIII, ICD–10) are highly frequent in the first studied sub-period in the town of Mali Lošinj. In the three studied sub-periods in the town of Mali Lošinj, a decreasing trend in the number of deaths caused by diseases classified within chapter *Diseases of the circulatory system* (chapter IX, ICD–10) is found. In the town of Bakar, an increase is found in the number of deaths by these causes, particularly in the third studied sub-period. In the first studied sub-period an almost threefold higher frequency of death is found caused by diseases classified within the *Diseases of the respiratory system* (chapter X, ICD–10) in the town of Mali Lošinj compared to the number of deaths in the town of Bakar in the same period. In the following periods their frequency is decreasing in both populations but is still higher in the town of Mali Lošinj. The number of deaths caused by diseases classified within chapter *External causes of morbidity and mortality* (chapter XX, ICD–10) in the studied periods has continually been decreasing in the town of Bakar while it increased in the area of the town of Mali Lošinj.

By comparing frequencies of the number of deceased in the towns of Bakar and Mali Lošinj according to causes of death classified within chapters of the Tenth Revision of the International Classification of Diseases, a statistically significant difference has been found (*p* < 0.01) for causes of death classified within chapters II, V, IX, X, XIII, XVIII and XX of the ICD–10.

Average age-standardised mortality rates from neoplasms show an increasing trend in both studied populations (Table 3). In the Bakar town area, in all three studied sub-periods, age-standardised rates of deaths from neoplasms are higher than in the town of Mali Lošinj. Average age-standardised mortality rate from malignant diseases for the area of the town of Mali Lošinj has almost doubled in the last studied period compared to the first studied period. The increase of average standardised mortality rate from malignant diseases in the area of the town of Bakar is less prominent. The average age-standardised mortality rate from causes of deaths classified within chapter *Mental and behavioural disorders* (chapter V, ICD–10) in the town of Bakar, for the first and second studied sub-period show significantly higher values compared to age-standardised mortality rates in the same sub-periods for the town of Mali Lošinj. In both studied populations average age-standardised mortality rates from *Diseases of the circulatory system* (chapter IX, ICD–10) in the second studied sub-period show higher values in comparison to the first and third studied sub-period. Average age-standardised mortality rates from the *Diseases of the respiratory system* (chapter X, ICD–10) show higher values for the town of Mali Lošinj in all three studied sub-periods compared to those found for the town of Bakar. In the area of the town of Bakar, the average age-standardised mortality rate is higher in the second studied sub-period, compared to the rate in the first and third studied sub-periods. Age-standardised mortality rates due to causes classified within chapter *External causes of morbidity and mortality* (chapter XX, ICD–10) in both studied populations are decreasing throughout the studied sub-periods. In the first two sub-periods they are higher for the town of Bakar compared to the town of Mali Lošinj.

### 3.4. Individual Leading Causes of Death

According to the ranking of 10 leading causes of death for the town of Bakar (Table 4) and the town of Mali Lošinj (Table 5), in both populations the first place is taken by *Ischemic heart diseases* (I20–I25) (Table S8), while *Cerebrovascular diseases* (I60–I69) occupy the second place. *Diseases of the myocardium/cardiomyopathies* (I40–I43) are in the third place among the leading causes of death in the area of the town of Bakar, while in the area of the town of Mali Lošinj this place is taken by diseases from the group *Heart failure, complications and ill-defined descriptions of heart disease* (I50–I52) (Table S9). The same group takes fourth place in the town of Bakar while in the town of Mali Lošinj *Chronic lower respiratory diseases* (J40–J47) are found in the fourth place. The fifth leading cause of death in the town of Bakar are *Organic, including symptomatic mental disorders* (F00–F09), while in the area of the town of Mali Lošinj this place is taken by the causes from the group *Other ill-defined and unspecified causes of mortality* (R99). Within both studied populations, the sixth place among the leading causes of death is taken by the group *Malignant neoplasm of trachea, bronchus and lung* (C33–C34), while *Diabetes mellitus* occupies eighth place (E10–E14). In the town of Mali Lošinj another unspecified cause of death ranked in the seventh place is *Senility* (R54), while in the town of Bakar ranked seventh among the leading causes of death is stomach carcinoma (C16) (Table S10). In the town of Mali Lošinj, *Pneumonia, organism unspecified* (J18) takes the second-last spot, while in the town of Bakar pneumonia is ranked last. In the town of Mali Lošinj, the last place among the leading causes of death is taken by causes classified among the group *Diseases of the myocardium/cardiomyopathy* (I40–I43).

The average age-standardised mortality rate from stomach cancer (C16) in all three studied sub-periods is higher for the town of Bakar than that for town of Mali Lošinj (Table 6). In both studied populations, the average age-standardised mortality rate from stomach cancer (C16) is highest in the first studied sub-period. The average age-standardised mortality rate from the group of organic, including symptomatic, mental disorders (F00–F09) in the first studied sub-period for the town of Bakar is 10 times higher than that for the town of Mali Lošinj. Approximately similar values for both towns are found only in the last studied sub-period. The highest average age-standardised mortality rates are found for diseases of the myocardium and cardiomyopathies (I40–I43) in the last studied sub-period in both populations. Average age-standardised mortality rates from the cause of death *Heart failure, complications and ill-defined descriptions of heart disease* (I50–I52) within the chapter *Diseases of the circulatory system* (chapter IX, ICD–10) are on the decline from the first studied sub-period onwards. In the first studied sub-period, the average age-standardised mortality rate is almost twice as higher in the town of Mali Lošinj compared to the town of Bakar. Average age-standardised mortality rates from chronic lower respiratory diseases (J40–J47) in all three studied sub-periods for the town of Mali Lošinj are higher than the average age-standardised rates for the town of Bakar. In the town of Mali Lošinj area, the average age-standardised mortality rate is on the decline between the second and third studied sub-periods, while in the area of the town of Bakar there is a rising trend since the first studied sub-period.

## 4. Discussion

This research represents the first systematic analysis of mortality characteristics of two selected populations in the northern Adriatic area for the period 1960–2012. During one third of the studied period, the town of Bakar has been subject to heavy industrial environment pollution. Industrial air pollution in the 1980s has marked the wider area of the macro-regional centre of the city of Rijeka, to which the town of Bakar also belongs. In spite of a general decrease of sulphur dioxide emissions in the mid 1990s, the results of a study conducted by Matković and Alebić–Juretić [10] have shown that such a decrease in emissions, following the closing of the coking plant, was found to be smaller at monitoring sites in the town of Bakar area compared to other monitoring sites in the wider area of the city of Rijeka. As a potential cause of such a decrease, meteorological conditions are cited, as is the direction of prevailing winds and the change in chemical composition of the local atmosphere following the closing of the coking plant.

For the entire studied period there were no significant deviations in the availability of archive sources with an exception for the years 1972, 1975 and 1976 for the town of Mali Lošinj. The reason for this was a fire in the archive storage in Mali Lošinj in 1981, in which part of the archived documentation was destroyed.

The average mortality rates of both populations through three studied sub-periods show trends of opposite directions: in the town of Bakar area there was an increase, and in the town of Mali Lošinj area a decline. An increasing trend of the average mortality rate in the town of Bakar can be explained by the transition between two mortality regimes which, aside from the Croatian territory, was also found in other European areas [38]. A decline of the average mortality rate in the town of Mali Lošinj can be explained by the process of depopulation of the Lošinj archipelago islands that had already started earlier, but also by the flow of young working population into the only urban centre on the island, the town of Mali Lošinj [16].

In the overall studied period, the coding of causes of death was regulated by several consecutive revised editions of the International Classification of Diseases and Related Health Problems. For the period prior to 1994, uncoded diagnoses from the reports on death were used and thus, in this way, the influence of changed coding on the analysis of the mortality characteristics among the two studied populations was reduced. This did not make it possible to remove the specificities of cause of death entries brought about by a physician or other responsible person [39].

In 2003, Croatia was ranked among countries with medium quality mortality statistics data [40]. A probable reason for this is its level of completion in a share between 70–90%, since already from the beginning of the 1990s, the criterion of < 10% of unidentified causes of death in the total annual mortality of Croatia was already fulfilled [41], which this study has confirmed for an even longer period of time.

Data on causes of deaths according to ICD–10 for the towns of Bakar and Mali Lošinj are comparable with data on causes of deaths for the Croatian level for the period 1985–2012 [36]. The ranking of the two leading causes of death according to ICD–10 at the Croatian level and the level of the studied communities for the period 1985–2012 is the same (chapter IX and chapter II, ICD–10).

A significant share of the causes of deaths classified within the chapter *Symptoms, signs and abnormal clinical and laboratory findings, not elsewhere classified* (chapter XVIII, ICD–10) was also frequent at the Croatian level for the major part of the studied period [38]. This was a result of several factors: the level and availability of health care [42,43], particularly for the islands, and the quality of reporting on the causes of death and mortality statistics maintenance.

In the Mali Lošinj town area, a particularly frequent cause of death from this group of diseases for the population residing on the smaller islands of the Lošinj archipelago gravitating towards the central island of Lošinj, is in part due to the fact that in these smaller islands there was no regular medical service, and reports on death were, for a part of the studied period, filed by health workers other than medical doctors.

A comparison of age-standardised mortality rates from neoplasms in the areas of the towns of Bakar and Mali Lošinj to the age-standardised rates for the Republic of Croatia for the period 1985–2012 (Figure S2), shows that the age-standardised rates for the areas of both towns were higher than the national level in 1986, 2001, 2003, 2005 and 2010. Age-standardised rates for the area of the town of Bakar were higher compared to the national level in 1992, 1993, 1995, and 2012 while in the town of Mali Lošinj this was the case in 1997, 2002, 2004 and 2007.

The areas where the studied populations lived are also areas of a region (Primorje-Gorski Kotar County) in which standardised rates of deaths from neoplasms can be followed from 1995. This mortality rate was higher from 1995–2000 in comparison to standardised mortality rate form neoplasms for the Croatian level and its renewed increase is found in the period 2003–2012 [44,45,46]. A significant rise of average standardised mortality rates for the area of the town of Mali Lošinj in the studied periods can be explained by increased availability of health care in the islands, an improved and more specialised diagnostics, and ultimately by a more developed system of coding the underlying cause of death. Among European countries, Croatia is placed in the top half according to age-standardised mortality rates from carcinoma of the gastrointestinal tract, with a decreasing tendency [47]. In both populations, we found that the mortality rate from stomach carcinoma also shows a declining tendency. In the aetiology of stomach carcinoma as a relevant factor, chronic infection of the mucous with *Helicobacter pylori* is noted [47]. According to results of the study on the prevalence of infection with *Helicobater pylori* in Croatia from 1988, Babuš V et al. have found that the prevalence of infection with *Helicobater pylori* in the south of Croatia statistically significantly differs, and is higher than in the central and northern part of Croatia [48]. Data on other etiological factors influencing the development of stomach carcinoma: type of diet [47,49,50,51,52], socioeconomic status, smoking [53,54,55] or exposure at the workplace [56,57,58,59] are not known for the respondents included in this study. This research could not determine whether the lower-than-average values in age-standardised mortality rates from stomach carcinoma among the population from the town of Mali Lošinj are a consequence of the protective influence of the Mediterranean diet [60,61].

In the area of the town of Bakar, a high average age-standardised rate for causes of deaths from the group *Organic, including symptomatic mental disorders* (chapter V, ICD–10) was found. In northern European countries during the 1980s and the 1990s there is a larger annual increase of mortality caused by dementia in the population from 80 years of age onwards [39,62]. In the overall studied period for causes of deaths classified within chapter V of the ICD–10, the share of diagnoses from the group *Organic, including symptomatic mental disorders* amounts to 93.48% for the town of Bakar and 50.88% for the town of Mali Lošinj. Such a difference in the frequency of a diagnosis from the group of *Organic, including symptomatic mental disorders* can, along with the result of diagnostic procedures development and availability, primarily be explained by specificities of filing the reports on deaths, which is supported by a significant decline in the number of entries for the town of Bakar in the last studied sub-period.

An increase of average age-standardised mortality rate from non-malignant respiratory diseases in the second studied sub-period in the area of the town of Bakar can be explained by the influence of air pollution on the increased mortality from this group of diseases. The relation of long-term effects of air pollution on mortality from respiratory diseases was also confirmed by other studies [63,64,65]. Among two studied communities, a difference has been determined in age-standardised mortality rates from chronic lower respiratory diseases. Within this group, chronic obstructive pulmonary disease (COPD) is present with a share of 81.08% and 71.68% in the towns of Bakar and Mali Lošinj, respectively. Since COPD is etiologically primarily linked to smoking [66], additional analysis is necessary that would, aside from smoking habit, also include the sources of indoor air pollution linked to worsening health status in persons suffering from COPD [67,68]. In the study on COPD prevalence among female respondents from the town of Bakar, it was determined that chronic bronchitis was equally present in the areas of the two settlements of the town of Bakar (Bakar and Krasica) as was in the control group that had not been exposed to significant air pollution (Viškovo) [9]. The same research among female non-smoking respondents in Bakar (measurements in 1986 and 1990) and Krasica (measurements in 1986), found lower values of forced vital capacity (FVC), forced expiratory volume in one second (FEV1) and the values of maximal expiratory flow between 25% and 75% of expired vital capacity (FMF 25–75%), in comparison to the control group (Viškovo), and the assumption is that lower lung function values could be a consequence of long-term air pollution [69]. In the study conducted by Žuškin et al., indoor pollution is considered a potential reason for a higher prevalence of chronic respiratory symptoms with respondents from the group of northern islands, compared to respondents from the group of southern islands, as part of a regional comparison of selected Croatian islands [70]. It was not possible to determine the long-term effects of air pollution on mortality from COPD in the studied area due to a small sample of the deceased and the absence of other relevant data (smoking, socioeconomic status).

Average age-standardised mortality rates from causes of deaths classified within the subgroup *Heart failure, complications and ill-defined descriptions of heart disease* (I50–I52) under the group *Other forms of heart diseases* in the chapter *Diseases of the circulatory system* (chapter IX, ICD–10) show a declining trend through the studied periods among both studied populations. Within this group, heart failure (I50) as a cause of death for the deceased from the town of Bakar in the overall studied period is present with a share of 81.98%, while in the town of Mali Lošinj its share amounts to 34.78%. Other causes of death from this subgroup relate to causes of death coded by I51 (*Complications and ill-defined descriptions of heart disease*). On the Croatian level, a declining mortality trend from cardiovascular diseases is present [71], as is the case with heart failure [72] which is confirmed by this study on the level of two populations of the littoral part of Croatia. A declining share of the causes of deaths coded by I51 in the area of the studied communities can be attributed to the development of diagnostics, availability, and higher level of adequate health care.

### Strengths and Limitations

Among the strengths of this study are also the large time period and sample size on which the mortality characteristics in the Croatian part of the northern Mediterranean region were studied. Another strength of this study is also the fact that for the studied period from 1960 to 1993 original archive documentation, that is, reports on death, were analysed. Those reports were recoded in the department for mortality statistics of the Croatian Institute of Public Health, the central national institution for determining and coding the underlying causes of death, the improvement of mortality statistics quality, and the compiling of the annual report on deceased persons in the Republic of Croatia. In this way, for this part of the studied period, the influence of different systems of coding the causes of death as consequences of several revisions of the International Classification of Diseases was brought down to the lowest possible level. Comparisons of frequencies of causes of death according to ICD–10 groups and average age-standardised rates have shown the existence of significant differences among the two studied populations, which opens the possibility of conducting further studies of individual causes of deaths and mortality predictions in this area.

A limitation of this study is that due to the unavailability of other data, such as data on smoking, lifestyle or socioeconomic status, that would enable additional conclusions about mortality characteristics within the studied communities, the study could not be designed in such a way as to have the features of a retrospective cohort study and surpass the framework of descriptive epidemiology. Thus, an ecological study has proved to be the most adequate design for this research. At the same time, the authors were aware of the fact that determining the confounding factor would be made difficult, and that one must take the ecological fallacy into account. A methodological limitation is also the fact that, for the period from 1994 to 2012, no original archive data were available and that instead of reports on death, the authors used the existing database of coded causes of deaths at the Teaching Institute of Public Health of Primorje-Gorski Kotar County. In this database, causes of deaths for those deceased in 1994 were coded according to ICD–9 and it was not possible to avoid a potential influence of various ways of determining and coding of underlying cause of death onto the discontinuity in the presentation of mortality characteristics of the two studied populations [36].

## 5. Conclusions

This study provides a systematic overview of the mortality characteristics for two populations in the littoral region of Croatia of which one had been exposed to industrial pollution. This study also contributes to the knowledge of mortality characteristics of a part of the Mediterranean, a significant area in the context of epidemiological studies of cardiovascular diseases and neoplasms. The study points to the problem of adequate reporting and filling in the reports on death. An increase of average age-standardised mortality rates from neoplasms in both studied populations, an increase in the average standardised mortality rates from respiratory diseases in the area exposed to industrial pollution, and the study of mortality from stomach carcinoma and COPD, particularly in the area of the town of Bakar, need additional investigation.

## Figures and Tables

**Table 1:** Population census and three age sub-groups according to census years 1961–2011 in the areas of the towns of Bakar and Mali Lošinj [24,25,26,27,28,29].

Town of Bakar						Town of Mali Lošinj				
Population	Age Groups				Census	Population	Age Groups			
	0–24	25–64	65+	Unknown Age			0–24	25–64	65+	Unknown Age
7788	2767	4 074	945	2	1961	8168	3 009	4024	1 130	5
8008	2821	4 024	1135	28	1971	6844	2 414	3292	1 101	37
7665	2485	3 799	1290	91	1981	7466	2 590	3661	1 134	81
7788	2422	4 228	1046	92	1991	8775	2 944	4731	1 005	95
7773	2227	4 326	1205	15	2001	8388	2 570	4535	1 240	43
8279	2020	4891	1368	0	2011	8116	2057	4722	1337	0

**Table 2:** Number of deaths and gender distribution according to periods in the towns of Bakar and Mali Lošinj according to cause of death classified within chapters of the International Classification of Diseases (ICD–10) chapters.

ICD Chapters of Diseases ICD–10 Code	Town of Bakar N /M:F/ (%)				Town of Mali Lošinj N /M:F/ (%)			
	Period							
	1960–1977	1978–1994	1995–2012	1960–2012	1960–1977	1978–1994	1995–2012	1960–2012
Infective and parasitic (A00–B99)	**28**	**6**	**6**	**40**	**21**	**16**	**6**	**43**
	**/19:9/**	**/5:1/**	**/3:3/**	**/27:13/**	**/12:9/**	**/8:8/**	**/3:3/**	**/23:20/**
	(1.86)	(0.42)	(0.39)	(0.89)	(1.50)	(1.24)	(0.43)	(1.05)
Neoplasms ^1^ (C00–D48)	**315**	**295**	**393**	**1003**	**160**	**224**	**377**	**761**
	**/175:140/**	**/172:123/**	**/217:176/**	**/564:439/**	**/88:72/**	**/127:97/**	**/203:174/**	**/418:343/**
	(20.94)	(20.80)	(25.35)	(22.43)	(11.45)	(17.42)	(26.74)	(18.59)
Blood, blood-forming organs (D50–D89)	**3**	**1**	**3**	**7**	**1**		**3**	**4**
	**/1:2/**	**/0:1/**	**/1:2/**	**/2:5/**	**/0:1/**		**/2:1/**	**/2:2/**
	(0.20)	(0.07)	(0.19)	(0.16)	(0.07)	**0**	(0.21)	(0.10)
Endocrine, metabolic system (E00–E90)	**26**	**58**	**49**	**133**	**36**	**59**	**37**	**132**
	**/2:24/**	**/15:43/**	**/20:29/**	**/37:96/**	**/10:26/**	**/22:37/**	**/10:27/**	**/42:90/**
	(1.73)	(4.09)	(3.16)	(2.97)	(2.58)	(4.59)	(2.62)	(3.23)
Mental and behavioural disorders ^1^ (F00–F99)	**130**	**90**	**10**	**230**	**20**	**20**	**17**	**57**
	**/32:98/**	**/27:63/**	**/8:2/**	**/67:163/**	**/8:12/**	**/13:7/**	**/10:7/**	**/31:26/**
	(8.64)	(6.35)	(0.65)	(5.14)	(1.43)	(1.56)	(1.21)	(1.39)
Nervous system (G00–G99)	**17**	**11**	**22**	**50**	**15**	**9**	**26**	**50**
	**/6:11/**	**/6:5/**	**/9:13/**	**/21:29/**	**/7:8/**	**/6:3/**	**/13:13/**	**/26:24/**
	(1.13)	(0.78)	(1.42)	(1.12)	(1.07)	(0.70)	(1.84)	(1.22)
Circulatory system ^1^ (I00–I99)	**690**	**696**	**769**	**2155**	**667**	**634**	**605**	**1906**
	**/286:404/**	**/310:386/**	**/332:437/**	**/928:1227/**	**/265:402/**	**/244:390/**	**/276:329/**	**/785:1121/**
	(45.88)	(49.08)	(49.61)	(48.19)	(47.75)	(49.30)	(42.91)	(46.57)
Respiratory system ^1^ (J00–J99)	**46**	**69**	**65**	**180**	**125**	**97**	**84**	**306**
	**/35:11/**	**/31:38/**	**/43:22/**	**/109:71/**	**/74:51/**	**/47:50/**	**/45:39/**	**/166:140/**
	(3.06)	(4.87)	(4.19)	(4.03)	(8.95)	(7.54)	(5.96)	(7.48)
Digestive system (K00–K93)	**49**	**49**	**43**	**141**	**40**	**62**	**43**	**145**
	**/29:20/**	**/29:20/**	**/27:16/**	**/85:56/**	**/18:22/**	**/36:26/**	**/20:23/**	**/74:71/**
	(3.26)	(3.46)	(2.77)	(3.15)	(2.86)	(4.82)	(3.05)	(3.54)
Skin, subcutaneous tissue (L00–L99)	**1**	**0**	**0**	**1**	**0**	**0**	**0**	**0**
	**/0:1/**			**/0:1/**				
	(0.07)			(0.02)				
Muscle, skeleton, connective tissue ^1^ (M00–M99)	**2**	**2**	**1**	**5**	**8**	**10**	**2**	**20**
	**/1:1/**	**/0:2/**	**/0:1/**	**/1:4/**	**/3:5/**	**/3:7/**	**/0:2/**	**/6:14/**
	(0.13)	(0.14)	(0.06)	(0.11)	(0.57)	(0.78)	(0.14)	(0.49)
Genitourinary system (N00–N99)	**12**	**10**	**18**	**40**	**16**	**12**	**26**	**54**
	**/6:6/**	**/5:5/**	**/10:8/**	**/21:19/**	**/10:6/**	**/6:6/**	**/15:11/**	**/31:23/**
	(0.80)	(0.71)	(1.16)	(0.89)	(1.15)	(0.93)	(1.84)	(1.32)
Pregnancy, childbirth, puerperium (O00–O99)	**0**	**0**	**0**	**0**	**2**	**0**	**0**	**2**
					(0.14)			(0.05)
							
Certain perinatal causes (P00–P96)	**23**	**12**	**7**	**42**	**19**	**5**	**11**	**35**
	**/14:9/**	**/8:4/**	**/3:4/**	**/25:17/**	**/9:10/**	**/3:2/**	**/7:4/**	**/19:16/**
	(1.53)	(0.85)	(0.45)	(0.94)	(1.36)	(0.39)	(0.78)	(0.86)
Congenital anomaly (Q00–Q99)	**7**	**9**	**2**	**18**	**9**	**6**	**3**	**18**
	**/3:4/**	**/6:3/**	**/2:0/**	**/11:7/**	**/3:5/**	**/2:4/**	**/1:2/**	**/6:11/**
	(0.47)	(0.63)	(0.13)	(0.40)	(0.64)	(0.54)	(0.21)	(0.46)
Symptoms, Ill–defined ^1^ (R00–R99)	**40**	**26**	**91**	**157**	**200**	**57**	**90**	**347**
	**/16:24/**	**/11:15/**	**/36:55/**	**/63:94/**	**/67:133/**	**/22:35/**	**/44:46/**	**/133:214/**
	(2.66)	(1.83)	(5.87)	(3.51)	(14.32)	(4.43)	(6.38)	(8.48)
External causes ^1^ (V01–Y98)	**115**	**84**	**71**	**270**	**58**	**74**	**80**	**212**
	**/74:41/**	**/52:32/**	**/47:24/**	**/173:97/**	**/32:26/**	**/40:34/**	**/45:35/**	**/117:95/**
	(7.65)	(5.92)	(4.58)	(6.04)	(4.15)	(5.75)	(5.67)	(5.18)
**Total**	1504	1418	1550	4472	1397	1286	1410	4092

^1^ marks ICD–10 chapters in which a statistically significant difference was found (Fischer’s exact test; *p* < 0.01).

**Table 3:** Average age-standardised mortality rates for the towns of Bakar and Mali Lošinj for the leading causes of death according to disease group in three studied sub-periods.

ICD Chapters of Diseases ICD–10 Code	Period	ASR/100,000 (World Health Organization (WHO))	
		Town	
		Bakar	Mali Lošinj
Neoplasms (C00–D48)	1960–1977	150.23	80.12
	1978–1994	152.37	113.83
	1995–2012	160.33	153.52
Mental and behavioural disorders (F00–F99)	1960–1977	58.23	9.05
	1978–1994	37.80	11.40
	1995–2012	3.88	6.28
Circulatory system (I00–I99)	1960–1977	305.62	291.47
	1978–1994	323.84	303.36
	1995–2012	295.26	232.46
Respiratory system (J00–J99)	1960–1977	20.29	57.04
	1978–1994	32.02	48.24
	1995–2012	24.40	33.02
External causes (V01–Y98)	1960–1977	73.28	45.56
	1978–1994	52.94	44.64
	1995–2012	34.58	38.74

**Table 4:** Ranking and share of 10 leading causes of death in the town of Bakar 1960–2012.

Rang	ICD–10 Code	Diagnosis	N	%
1	I20–I25	Ischemic heart diseases	847	18.94
2	I60–I69	Cerebrovascular diseases	527	11.78
3	I40–I43	Diseases of the myocardium/cardiomyopathy	239	5.34
4	I50–I52	Heart failure, complication, ill-defined	222	4.96
5	F00–F09	Organic, including symptomatic mental disorders	215	4.81
6	C33–C34	Malignant neoplasm of trachea, bronchus and lung	203	4.54
7	C16	Malignant neoplasm of stomach	137	3.06
8	E10–E14	Diabetes mellitus	124	2.77
9	I70	Atherosclerosis	118	2.64
10	J18	Pneumonia, organism unspecified	95	2.12

**Table 5:** Ranking and share of 10 leading causes of death in the town of Mali Lošinj 1960–2012.

Rang	ICD–10 Code	Diagnosis	N	%
1	I20–I25	Ischemic heart diseases	718	17.54
2	I60–I69	Cerebrovascular diseases	507	12.39
3	I50–I52	Heart failure, complication, ill-defined	368	8.99
4	J40–J47	Chronic lower respiratory diseases	173	4.23
5	R99	Other ill-defined and unspecified causes of mortality	158	3.86
6	C33–C34	Malignant neoplasm of trachea, bronchus and lung	151	3.69
7	R54	Senility	147	3.59
8	E10–E14	Diabetes melitus	132	3.23
9	J18	Pneumonia, organism unspecified	105	2.57
10	I40–I43	Diseases of the myocardium/cardiomyopathy	99	2.42

**Table 6:** Average age-standardised mortality rates for the towns of Bakar and Mali Lošinj according to leading causes of deaths in three studied sub-periods.

ICD–10 Code	Period	ASR/100,000 (WHO)	
		Town	
		Bakar	Mali Lošinj
Stomach cancer (C16)	1960–1977	31.32	12.64
	1978–1994	19.45	6.85
	1995–2012	11.66	9.17
Organic, symptomatic, mental disorders (F00–F09)	1960–1977	55.28	5.35
	1978–1994	34.52	4.16
	1995–2012	1.67	2.42
Myocardium, cardiomyopathy (I40–I43)	1960–1977	2.27	4.75
	1978–1994	7.49	4.52
	1995–2012	83.72	29.57
Heart failure, complication, ill-defined (I50–I52)	1960–1977	56.42	109.55
	1978–1994	26.10	35.63
	1995–2012	12.15	10.55
Chronic lower respiratory diseases (J40–J47)	1960–1977	9.42	30.29
	1978–1994	10.21	30.71
	1995–2012	11.46	15.54

## Supplementary Materials

The following are available online at http://www.mdpi.com/1660-4601/15/11/2591/s1, Figure S1. Average annual concentration of SO_2_ at air quality monitoring stations in the area of the town of Bakar in the period 1977–2012. For the year 1977 data are available from April to December (MV= marginal values of average annual SO_2_ concentration); Figure S2. Age-standardised rates from *Neoplasms* for the areas of the towns of Bakar and Mali Lošinj and for the Croatian level in the period 1985–2012/100,000 population; Table S1. Number of deaths, general mortality rate/100,000 population and age-standardised mortality rate/100,000 population in the towns of Bakar and Mali Lošinj for the period 1960–2012; Table S2. Average number of deaths, average general mortality rate/100,000 population and average age-standardised mortality rate/100,000 population in the towns of Bakar and Mali Lošinj in the various exposure periods; Table S3. Age-standardised mortality rate/100,000 population for the leading causes of deaths by disease group in the towns of Bakar and Mali Lošinj for the period 1960–2012; Table S4. Age-standardised mortality rate/100,000 population for the leading causes of deaths in the towns of Bakar and Mali Lošinj for the period 1960–2012; Table S5. Number of deaths and proportionate mortality for *Neoplasms* (C00–D48) in the towns of Bakar and Mali Lošinj for the period 1960–2012; Table S6. Proportionate mortality for *Mental and behavioural disorders* (F00–F99) in the towns of Bakar and Mali Lošinj for the period 1960–2012; Table S7. Proportionate mortality for *Diseases of the respiratory system* (J00–J99) in the towns of Bakar and Mali Lošinj for the period 1960–2012; Table S8. Proportionate mortality for *Diseases of the circulatory system* (I00–I99) in the towns of Bakar and Mali Lošinj for the period 1960–2012; Table S9. Proportionate mortality for *Other forms of heart disease* (I30–I52) in the towns of Bakar and Mali Lošinj for the period 1960–2012; Table S10. Proportionate mortality for *Malignant neoplasms of digestive organs* (C15–C26) in the towns of Bakar and Mali Lošinj for the period 1960–2012.
