# Supplementary material for: Mortality Characteristics of Two Populations in the Northern Mediterranean (Croatia) in the Period 1960–2012: An Ecological Study
Source: Int J Environ Res Public Health. 2018 Nov 20;15(11):2591. doi: 10.3390/ijerph15112591 (PMC6266380; doi:10.3390/ijerph15112591)
Supplement: Supplementary file 1 [file ijerph-15-02591-s001.pdf]

International Journal of
Environmental Research and Public Health

MDPI

Supplementary Material: Mortality Characteristics of Two Populations in the Northern Mediterranean (Croatia) in the Period 1960–2012: An Ecological Study

Robert Doričić, Tanja Ćorić, Morana Tomljenović, Danijela Lakošeljac, Amir Muzur and Branko Kolarić

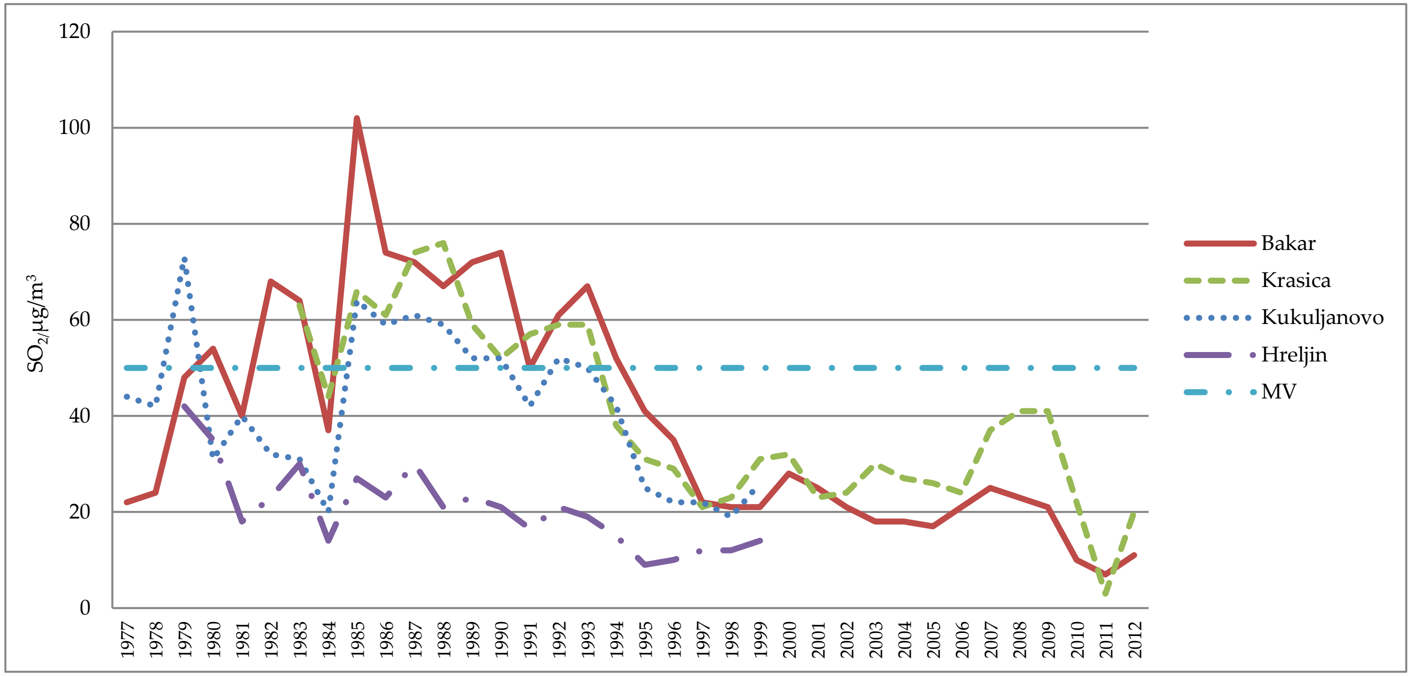

Figure S1. Average annual concentration of SO_2 at air quality monitoring stations in the area of the town of Bakar in the period 1977–2012. For the year 1977 data are available from April to December (MV= marginal values of average annual SO_2 concentration).

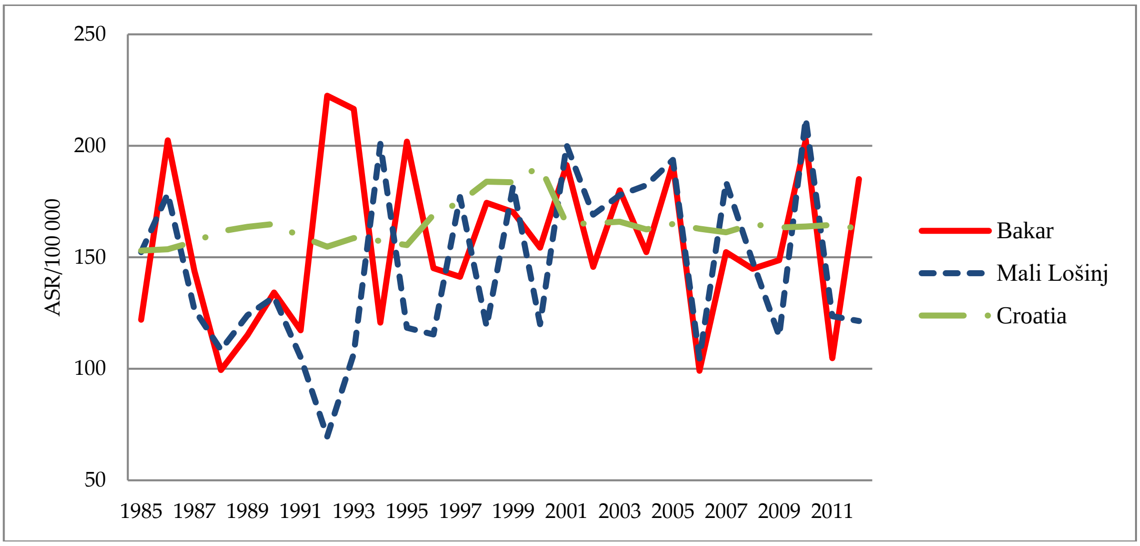

Figure S2. Age-standardised rates from *Neoplasms* for the areas of the towns of Bakar and Mali Lošinj and for the Croatian level in in the period 1985–2012/100,000 population.

Table S1. Number of deaths, general mortality rate/100,000 population and age-standardised mortality rate/100 000 population in the towns of Bakar and Mali Lošinj for the period 1960–2012.

Year	Deaths/N		GMR/100,000		ASR/100,000 (WHO)	
	Bakar	Mali Lošinj	Bakar	Mali Lošinj	Bakar	Mali Lošinj
1960	64	106	821.99	1298.54	601.07	874.62
1961	64	111	821.99	1359.79	645.84	927.11
1962	69	106	886.21	1298.54	638.36	1011.57
1963	72	79	924.74	967.78	672.75	635.45
1964	93	94	1 194.45	1151.54	890.55	760.25
1965	97	66	1 245.83	808.53	922.67	572.47
1966	91	81	1 140.35	1189.95	787.85	676.67
1967	85	68	1 065.16	998.97	747.05	627.18
1968	87	78	1 090.23	1145.88	723.81	704.26
1969	111	83	1 390.98	1219.33	904.92	725.30
1970	89	83	1 115.29	1219.33	736.80	764.87
1971	109	74	1 365.91	1087.12	926.66	644.95
1972	70	25	877.19	367.27	631.17	200.07
1973	86	103	1 077.69	1513.15	757.31	915.23
1974	76	95	952.38	1395.62	639.59	792.85
1975	83	30	1 040.10	440.72	702.62	264.22
1976	78	26	1 029.84	352.06	566.63	213.81
1977	80	89	1 056.25	1 205.15	620.96	738.14
1978	91	74	1 201.48	1 002.03	797.23	640.56
1979	92	68	1 214.68	920.79	721.91	556.02
1980	103	73	1 359.92	988.49	813.95	606.96
1981	91	82	1 201.48	1 110.36	713.95	700.29
1982	84	73	1 109.06	988.49	674.43	577.03
1983	63	82	831.79	1 110.36	500.92	748.97
1984	76	83	1 003.43	1 123.90	600.96	669.97
1985	72	82	950.62	1 110.36	632.42	736.24

1986	69	80	896.57	921.66	667.96	672.64
1987	86	74	1 117.46	852.53	762.11	611.86
1988	78	83	1 013.51	956.22	713.55	724.91
1989	74	79	961.54	910.14	624.95	675.50
1990	89	80	1 156.44	921.66	790.41	715.98
1991	84	74	1 091.48	852.53	752.67	635.75
1992	83	66	1 078.48	760.37	758.09	569.05
1993	94	64	1 221.41	737.33	787.54	563.95
1994	89	69	1 156.44	794.93	793.09	610.15
1995	91	60	1 182.43	691.24	805.00	544.91
1996	95	58	1 224.54	695.03	696.83	423.29
1997	71	61	915.18	730.98	571.59	465.23
1998	91	71	1 172.98	850.81	688.22	524.58
1999	100	96	1 288.99	1 150.39	752.98	780.16
2000	90	72	1 160.09	862.79	676.70	603.06
2001	89	83	1 147.20	994.61	669.56	648.47
2002	91	83	1 172.98	994.61	719.99	622.56
2003	77	82	992.52	982.62	606.07	613.08
2004	69	88	889.40	1 054.52	579.55	659.25
2005	88	90	1 134.31	1 078.49	700.25	650.22
2006	60	53	724.73	653.03	383.21	362.94
2007	99	90	1 195.80	1 108.92	623.51	612.99
2008	91	98	1 099.17	1 207.49	574.06	647.29
2009	90	75	1 087.09	924.10	576.36	522.74
2010	109	91	1 316.58	1 121.24	775.50	628.76
2011	62	81	748.88	998.03	401.91	514.93
2012	87	78	1 050.85	961.06	551.02	514.23

Table S2. Average number of deaths, average general mortality rate/100,000 population and average age-standardised mortality rate/100,000 population in the towns of Bakar and Mali Lošinj in the various exposure periods.

Period	Deaths/N		GMR/100,000		ASR/100,000 (WHO)	
	**Bakar**	**Mali Lošinj**	**Bakar**	**Mali Lošinj**	**Bakar**	**Mali Lošinj**
1960–1977	83.56	77.61	293.58	989.67	728.70	669.39
1978–1994	83.41	75.65	214.15	623.83	712.13	647.99
1995–2012	86.11	78.33	237.26	574.38	630.68	574.37

Table S3. Age-standardised mortality rate/100 000 population for the leading causes of deaths by disease group in the towns of Bakar and Mali Lošinj for the period 1960–2012.

ASR/100,000 (WHO)										
**ICD Chapters of diseases/ ICD–10 code**	**Neoplasms (C00–D48)**		**Mental and behavioural disorders (F00–F99)**		**Circulatory system (I00–I99)**		**Respiratory system (J00–J99)**		**External causes (V01–Y98)**	
	**Bakar**	**Mali Lošinj**	**Bakar**	**Mali Lošinj**	**Bakar**	**Mali Lošinj**	**Bakar**	**Mali Lošinj**	**Bakar**	**Mali Lošinj**
1960	104.36	91.66	156.53	29.09	197.57	381.28	17.39	108.80	48.30	57.11
1961	177.19	76.21	52.18	0.00	148.60	449.79	26.09	86.29	48.05	7.27
1962	160.14	83.85	147.84	7.27	201.01	268.61	0.00	29.09	24.33	332.93
1963	101.14	93.44	78.27	0.00	323.51	216.12	8.70	45.62	70.56	14.55
1964	182.28	60.86	78.27	9.95	250.49	379.04	17.39	110.25	81.35	7.27
1965	144.47	86.50	113.05	0.00	351.11	172.22	35.77	44.79	67.17	27.92
1966	214.33	66.02	36.20	13.33	273.73	289.42	14.48	52.25	73.10	22.83
1967	130.43	93.54	21.72	13.74	314.43	232.62	50.42	90.45	132.91	27.14
1968	173.78	107.98	16.30	7.46	348.20	245.60	32.59	88.16	72.97	26.90
1969	169.90	101.94	43.44	52.25	436.29	266.40	21.72	67.18	89.23	59.70
1970	187.97	93.33	36.20	7.46	303.16	346.56	43.63	96.24	63.41	28.44
1971	106.75	96.32	28.96	0.00	496.18	304.09	23.54	66.21	108.22	51.62
1972	100.63	15.37	48.81	0.00	319.19	110.77	0.00	7.46	78.65	0.00
1973	155.27	119.52	29.15	14.93	363.00	463.31	14.48	59.84	99.46	97.83
1974	85.58	103.97	7.24	7.46	384.50	482.09	0.00	37.76	75.04	7.46
1975	215.52	22.39	21.72	0.00	292.84	119.87	14.48	7.46	81.73	0.00
1976	136.95	21.74	70.08	0.00	252.86	86.96	19.11	0.00	63.05	0.00
1977	157.45	107.55	62.16	0.00	244.51	431.64	25.48	28.99	41.56	51.18
1978	203.32	89.34	25.48	0.00	359.24	274.32	30.30	105.47	86.57	96.20

1979	162.16	65.22	63.71	7.25	260.29	264.44	29.51	26.59	72.63	53.55
1980	121.76	41.00	82.82	0.00	299.14	372.82	19.11	61.92	123.23	32.54
1981	168.21	115.95	86.17	12.10	318.57	270.17	25.48	71.25	32.56	33.00
1982	181.53	129.04	57.33	7.25	270.56	231.90	37.32	59.19	32.29	7.25
1983	97.92	113.96	49.41	37.86	203.92	290.58	50.96	36.23	23.68	31.90
1984	161.09	76.02	57.33	27.04	268.89	276.46	25.48	59.52	43.56	63.88
1985	121.94	152.39	31.85	0.00	311.36	311.32	43.04	56.03	55.05	111.16
1986	202.46	178.42	0.00	8.18	248.57	310.73	44.66	49.06	82.15	14.54
1987	143.96	126.54	23.57	0.00	418.37	303.85	26.36	32.71	27.55	40.88
1988	99.52	108.55	23.57	16.35	338.27	303.28	40.69	65.42	49.93	54.61
1989	115.14	123.95	15.71	23.35	302.52	308.34	47.14	57.24	37.31	27.56
1990	134.19	132.20	0.00	17.30	415.92	315.14	43.26	50.01	46.97	82.42
1991	117.29	105.18	23.57	12.64	402.17	388.29	7.86	8.18	74.22	46.58
1992	222.47	69.64	23.57	0.00	381.09	379.45	7.86	8.18	16.23	28.60
1993	216.58	106.81	31.43	0.00	373.24	277.79	15.71	48.54	21.79	34.16
1994	120.73	200.85	47.14	24.53	333.11	278.24	49.71	24.53	74.24	0.00
1995	201.84	118.47	0.00	0.00	355.84	234.46	48.54	24.53	16.18	50.73
1996	145.19	115.36	0.00	0.00	368.67	152.60	20.46	49.12	42.40	18.71
1997	141.25	177.03	0.00	6.63	243.97	200.29	28.74	13.25	26.15	15.00
1998	174.38	118.99	0.00	9.35	320.77	265.93	13.64	35.86	65.07	24.55
1999	170.31	181.88	13.64	0.00	334.56	276.29	49.50	123.05	38.40	42.01
2000	154.35	119.96	0.00	0.00	371.84	295.24	20.46	19.88	60.52	41.84
2001	191.40	200.29	6.82	6.63	341.62	231.28	35.56	19.88	31.15	35.86
2002	145.64	169.14	0.00	0.00	358.42	300.13	27.28	39.76	64.12	52.53
2003	179.99	177.92	0.00	6.63	275.80	255.91	8.58	19.88	38.70	51.70
2004	152.26	182.33	0.00	0.00	226.27	233.06	13.64	26.51	35.86	53.64
2005	191.76	193.69	19.33	21.76	303.17	252.55	22.22	39.76	59.69	44.21
2006	99.12	104.56	6.01	0.00	169.04	190.18	18.02	6.15	6.01	28.83
2007	152.37	183.78	0.00	18.98	278.09	180.20	36.04	64.79	24.38	45.89

2008	144.79	148.12	6.01	0.00	258.88	264.94	36.04	24.59	13.92	73.97
2009	148.79	114.65	6.01	6.15	287.57	170.80	36.04	38.23	29.97	30.73
2010	202.62	212.17	0.00	0.00	352.93	235.20	12.50	12.29	40.99	27.15
2011	104.80	123.58	0.00	30.73	194.66	238.55	6.01	18.44	5.94	25.14
2012	185.10	121.47	12.01	6.15	272.65	206.65	6.01	18.44	22.97	34.78

Table S4. Age-standardised mortality rate/100,000 population for the leading causes of deaths in the towns of Bakar and Mali Lošinj for the period 1960–2012.

	ASR/100,000 (WHO)									
**ICD–10 code**	**Stomach cancer (C16)**		**Organic symptomatic mental disorders (F00–F09)**		**Myocardium, cardiomyopathy (I40–I43)**		**Heart failure, complication, ill-defined (I50–I52)**		**Chronic lower respiratory diseases (J40–J47)**	
	**Bakar**	**Mali Lošinj**	**Bakar**	**Mali Lošinj**	**Bakar**	**Mali Lošinj**	**Bakar**	**Mali Lošinj**	**Bakar**	**Mali Lošinj**
1960	8.70	21.82	156.53	29.09	0.00	0.00	86.97	130.99	8.70	65.09
1961	26.09	21.45	52.18	0.00	0.00	0.00	52.18	261.52	17.39	36.36
1962	43.49	7.27	147.84	0.00	0.00	0.00	43.48	181.81	0.00	7.27
1963	26.09	27.03	78.27	0.00	0.00	0.00	0.00	87.27	8.70	16.53
1964	35.55	7.27	78.27	0.00	14.17	0.00	43.48	154.60	8.70	75.41
1965	26.09	0.00	113.05	0.00	14.17	0.00	60.88	72.73	17.39	24.50
1966	51.06	7.46	36.20	0.00	0.00	0.00	72.41	52.25	0.00	44.78
1967	0.00	7.46	21.72	0.00	0.00	0.00	72.41	22.39	21.72	45.23
1968	41.57	13.33	7.24	7.46	0.00	0.00	63.43	80.95	0.00	44.78
1969	65.16	15.37	43.44	44.78	0.00	0.00	28.96	82.11	14.48	44.78
1970	61.24	7.46	36.20	7.46	0.00	0.00	56.19	127.33	28.96	35.96
1971	7.24	22.83	28.96	0.00	12.61	0.00	92.44	126.89	16.30	30.30
1972	14.48	0.00	36.20	0.00	0.00	0.00	57.92	59.71	0.00	0.00
1973	21.91	15.37	21.72	7.46	0.00	7.46	98.05	89.57	7.24	22.83
1974	36.20	22.83	7.24	0.00	0.00	7.46	50.68	178.66	0.00	22.39
1975	40.83	14.93	21.72	0.00	0.00	0.00	72.41	74.64	7.24	0.00

1976	19.83	0.00	70.08	0.00	0.00	0.00	38.22	72.47	6.37	0.00
1977	38.22	15.71	38.22	0.00	0.00	70.52	25.48	115.95	6.37	28.99
1978	44.41	7.25	19.11	0.00	12.74	28.99	71.52	65.22	0.00	76.49
1979	25.48	0.00	63.71	7.25	18.21	7.25	19.11	79.71	0.00	26.59
1980	12.74	0.00	76.45	0.00	0.00	0.00	32.57	21.74	6.37	25.29
1981	0.00	0.00	68.53	0.00	19.55	0.00	25.48	51.94	12.74	34.29
1982	18.21	7.25	57.33	0.00	19.83	7.25	36.58	21.74	12.74	28.99
1983	36.67	0.00	31.85	7.25	6.37	0.00	19.11	14.49	12.74	21.74
1984	30.30	7.25	57.33	7.25	0.00	0.00	12.74	32.54	0.00	59.52
1985	37.32	7.25	31.85	0.00	11.84	15.71	36.67	14.49	23.93	34.29
1986	26.66	16.35	0.00	0.00	7.86	0.00	15.71	32.71	9.26	40.89
1987	15.71	9.47	23.57	0.00	17.02	8.18	23.57	32.71	10.65	32.71
1988	18.29	8.18	23.57	8.18	13.93	0.00	23.57	32.71	17.12	40.89
1989	15.71	8.18	15.71	8.18	0.00	0.00	0.00	24.53	7.86	16.35
1990	24.09	24.53	0.00	8.18	0.00	0.00	15.71	32.71	10.43	25.48
1991	9.26	12.64	23.57	0.00	0.00	9.48	7.86	40.89	7.86	8.18
1992	15.71	0.00	15.71	0.00	0.00	0.00	7.86	42.18	0.00	0.00
1993	0.00	8.18	31.43	0.00	0.00	0.00	78.57	49.06	7.86	34.00
1994	0.00	0.00	47.14	24.53	0.00	0.00	17.12	16.35	34.00	16.35
1995	0.00	0.00	0.00	0.00	62.85	41.83	15.71	8.18	15.71	8.18
1996	20.46	0.00	0.00	0.00	170.50	13.25	0.00	6.63	0.00	35.86
1997	15.10	16.57	0.00	0.00	95.48	39.76	0.00	0.00	0.00	6.63
1998	0.00	9.35	0.00	0.00	152.12	33.14	6.82	33.14	0.00	29.24
1999	6.82	9.35	0.00	0.00	144.98	76.76	75.02	13.25	35.86	58.76
2000	16.05	6.63	0.00	0.00	150.04	33.14	27.28	13.25	6.82	6.63
2001	30.19	6.63	0.00	0.00	143.22	26.51	8.28	6.63	20.46	13.25
2002	6.82	25.13	0.00	0.00	88.66	6.63	0.00	6.63	13.64	19.88
2003	0.00	6.63	0.00	0.00	68.20	26.51	6.82	6.63	8.58	13.25
2004	25.70	6.63	0.00	0.00	49.50	39.76	6.82	19.88	0.00	6.63

2005	0.00	7.98	0.00	6.63	49.20	53.02	0.00	19.88	8.58	19.88
2006	12.50	6.15	6.01	0.00	12.01	0.00	0.00	6.15	6.01	6.15
2007	18.02	6.15	0.00	0.00	66.08	43.03	23.82	0.00	12.01	12.29
2008	20.41	18.44	6.01	0.00	54.07	49.17	6.01	6.15	24.03	6.15
2009	13.85	6.15	6.01	6.15	60.07	6.15	18.02	6.15	36.04	12.29
2010	6.01	20.38	0.00	0.00	30.04	0.00	12.01	25.14	12.50	0.00
2011	6.01	0.00	0.00	30.73	6.01	12.84	6.01	12.29	6.01	6.15
2012	11.88	12.84	12.01	0.00	104.03	30.73	6.01	0.00	0.00	18.44

Table S5. Number of deaths and proportionate mortality for *Neoplasms* (C00–D48) in the towns of Bakar and Mali Lošinj for the period 1960–2012.

**ICD Chapters of diseases ICD–10 code**	**Town of Bakar N (%)**				**Town of Mali Lošinj N (%)**			
	Period							
	1960–1977	1978–1994	1995–2012	1960–2012	1960–1977	1978–1994	1995–2012	1960–2012
Lip, oral cavity and pharynx (C00–C14)	4	4	12	20 (1.99)	3	2	12	17 (2.11)
Digestive organs (C15–C26)	148	103	123	374 (37.25)	63	87	105	255 (33.55)
Respiratory and intrathoracic organs (C30–C39)	43	75	106	224 (1.73)	29	52	89	170 (4.09)
Bone and articular cartilage (C40–C41)	3	3	4	10 (1.00)	4	4	1	9 (1.18)
Melanoma and other malignant neoplasms of skin (C43–C44)	2	5	15	22 (2.19)	0	3	5	8 (1.05)
Mesothelial and soft tissue (C45–C49)	9	3	6	18 (1.79)	1	3	2	6 (0.79)
Breast (C50–C50)	22	15	27	64 (6.37)	7	14	36	57 (7.50)
Female genital organs (C51–C58)	15	15	23	53 (5.28)	15	11	15	41 (5.39)
Male genital organs (C60–C63)	19	15	17	51 (5.08)	8	14	26	48 (6.32)
Urinary tract (C64–C68)	14	7	12	33 (3.29)	12	10	12	34 (4.47)
Eye, brain and other parts of central nervous system (C69–C72)	10	11	15	36 (3.59)	3	8	15	26 (3.42)
Thyroid and other endocrine glands (C73–C75)	1	1	1	3 (0.30)	1	1	3	5 (0.66)
Ill-defined, secondary and unspecified sites (C76–C80)	13	16	16	45 (4.48)	10	7	41	58 (7.63)
Stated or presumed to be primary of lymphoid, haematopoietic and related tissue (C81–C96)	8	9	17	34 (3.39)	1	2	18	21 (2.76)
Benign neoplasms (D10–D36)	6	10	0	16 (1.59)	1	4	0	5 (0.66)
Neoplasms of uncertain or unknown behaviour (D37–D48)	0	1	0	1 (0.10)	0	1	0	1 (0.13)

Table S6. Proportionate mortality for *Mental and behavioural disorders* (F00–F99) in the towns of Bakar and Mali Lošinj for the period 1960–2012.

Mental and behavioural disorders (F00–F99)	Bakar (%)	Mali Lošinj (%)
Organic, including symptomatic, mental disorders (F00–F09)	93.48	50.88
Mental and behavioural disorders due to psychoactive substance use (F10–F19)	5.22	40.35
Schizophrenia, schizotypal and delusional disorders (F20–F29)	1.30	3.51
Mood [affective] disorders (F30–F39)	0	3.51
Unspecified mental disorder (F99–F99)	0	1.75

Table S7. Proportionate mortality for *Diseases of the respiratory system* (J00–J99) in the towns of Bakar and Mali Lošinj for the period 1960–2012.

Diseases of the respiratory system (J00–J99)	Bakar (%)	Mali Lošinj (%)
Influenza and pneumonia (J09–J18)	57.22	40.66
Other acute lower respiratory infections (J20–J22)	0	0.33
Chronic lower respiratory diseases (J40–J47)	41.11	57.38
Other respiratory diseases principally affecting the interstitium (J80–J84)	0	0.98
Suppurative and necrotic conditions of lower respiratory tract (J85–J86)	1.11	0
Other diseases of pleura (J90–J94)	0	0.33
Other diseases of the respiratory system (J95–J99)	0.56	0.33

Table S8. Proportionate mortality for *Diseases of the circulatory system* (I00–I99) in the towns of Bakar and Mali Lošinj for the period 1960–2012.

Diseases of the circulatory system (I00–I99)	Bakar (%)	Mali Lošinj (%)
Chronic rheumatic heart diseases (I05–I09)	0.19	0.26
Hypertensive diseases (I10–I15)	0.46	0.94
Ischemic heart diseases (I20–I25)	39.32	37.63
Pulmonary heart disease and diseases of pulmonary circulation (I26–I28)	3.48	1.94
Other forms of heart disease (I30–I52)	25.53	26.94
Cerebrovascular diseases (I60–I69)	24.47	26.57
Diseases of arteries, arterioles and capillaries (I70–I79)	6.36	5.35
Diseases of veins, lymphatic vessels and lymph nodes, not elsewhere classified (I80–I89)	0.19	0.37

Table S9. Proportionate mortality for *Other forms of heart disease* (I30–I52) in the towns of Bakar and Mali Lošinj for the period 1960–2012.

**Other forms of heart disease (I30–I52)**	**Bakar (%)**	**Mali Lošinj (%)**
Pericardium (I30–I32)	0.18	0
Endocardium, valve disorders (I33–I39)	3.09	1.36
Myocardium, cardiomyopathy (I40–I43)	44.00	19.65
Rhythm and conduction disorders (I44–I49)	12.36	7.39
Heart failure, complication, ill-defined (I50–I52)	40.36	71.60

Table S10. Proportionate mortality for *Malignant neoplasms of digestive organs* (C15–C26) in the towns of Bakar and Mali Lošinj for the period 1960–2012.

**Malignant neoplasms of digestive organs (C15–C26)**	**Bakar (%)**	**Mali Lošinj (%)**
Oesophagus (C15)	4.01	2.75
Stomach (C16)	36.63	25.10
Small intestine (C17)	1.07	0.39
Colon, rectosigmoid junction, rectum (C18–C20)	24.06	32.94
Liver, intrahepatic bile ducts (C22)	12.30	12.94
Gallbladder (C23)	1.34	0.78
Other and unspecified parts of biliary tract (C24)	1.60	1.18
Pancreas (C25)	9.63	12.16
Other and ill-defined digestive organs (C26)	9.36	11.76
